# Phase relaxed localized excitation pulses for inner volume fast spin echo imaging

**DOI:** 10.1002/mrm.25996

**Published:** 2015-10-09

**Authors:** Shaihan J. Malik, Joseph V. Hajnal

**Affiliations:** ^1^ Biomedical Engineering Department, Division of Imaging Sciences, King's College LondonSt. Thomas' HospitalLondonUnited Kingdom

**Keywords:** RF pulse design, FSE, inner volume imaging, parallel transmission, phase relaxation, CPMG, magnitude least squares

## Abstract

**Purpose:**

To design multidimensional spatially selective radiofrequency (RF) pulses for inner volume imaging (IVI) with three‐dimensional (3D) fast spin echo (FSE) sequences. Enhanced background suppression is achieved by exploiting particular signal properties of FSE sequences.

**Theory and Methods:**

The CPMG condition dictates that echo amplitudes will rapidly decrease if a 90° phase difference between excitation and refocusing pulses is not present, and refocusing flip angles are not precisely 180°. This mechanism is proposed as a means for generating additional background suppression for spatially selective excitation, by biasing residual excitation errors toward violating the CPMG condition. 3D RF pulses were designed using this method with a 3D spherical spiral trajectory, under‐sampled by factor 5.6 for an eight‐channel PTx system, at 3 Tesla.

**Results:**

3D‐FSE IVI with pulse durations of approximately 12 ms was demonstrated in phantoms and for T_2_‐weighted brain imaging in vivo. Good image quality was obtained, with mean background suppression factors of 103 and 82 ± 6 in phantoms and in vivo, respectively.

**Conclusion:**

Inner Volume Imaging with 3D‐FSE has been demonstrated in vivo with tailored 3D‐RF pulses. The proposed design methods are also applicable to 2D pulses. Magn Reson Med 76:848–861, 2016. © 2015 The Authors. Magnetic Resonance in Medicine published by Wiley Periodicals, Inc. on behalf of International Society for Magnetic Resonance in Medicine

## INTRODUCTION

In a typical MRI examination, the majority of spatial encoding is achieved during signal reception, with the role of the excitation limited to localize within a slice or slab. In some cases, it is however more convenient to localize signal from a more generally shaped region and, hence, to push some of the burden of spatial encoding to the excitation. So‐called inner volume imaging (IVI) has existed since the early days of MRI 1 as a means for imaging only a small region embedded within a larger object, and for suppression of motion artifacts originating outside the region of interest. Inner volume selection can be achieved using a combination of 1D selective pulses 1 or a single multidimensional selective pulse 2. Multidimensional pulses are convenient in that they may be incorporated into a wide range of sequences, but have the drawback of typically long durations necessary to achieve spatial localization by gradient encoding, particularly if three‐dimensional (3D) selective excitations are required. This problem can be reduced by using parallel transmission (PTx) to accelerate the pulses, and has been demonstrated for 3D selection in animals 3 and humans 4.

Multidimensional pulses are particularly well suited for IVI using spin echo or fast spin echo (FSE) sequences because magnetization not tipped by the initial excitation pulse does not give signal at any of the subsequent echo times so long as sufficient spoiling is included to suppress free induction decay (FID) signals produced by refocusing pulses. This property, a consequence of the structure and timing of the sequence, is true whether the refocusing pulses are selective or nonselective, irrespective of flip angle. Inner volume selection achieved upon excitation is therefore maintained throughout a long echo train even if nonselective refocusing pulses are used (i.e., 3D‐FSE) and even if the refocusing angles are less than 180° 5. A good general description of the properties of 3D‐FSE sequences including a discussion of FID spoiling is given by Mugler 6. Mitsouras et al 5 also give a detailed analysis of salient features regarding inner volume imaging. The 3D‐FSE IVI has been used with 2D selective excitations to image blood vessels 7, 8, prostate 9 and recently, the orbits 10.

Background suppression is critical for inner‐volume imaging, particularly when selecting a small sub‐volume since the volume contributing undesired background signals is large. This work proposes a modified pulse design method specifically tailored for producing enhanced background suppression in FSE sequences with long echo trains and low refocusing angles, with particular focus on 3D‐FSE. The method is combined with a VERSE based procedure for minimizing peak power (i.e., maximum amplitude) of the resulting pulses and used for on‐line design of 3D spatially selective PTx excitation pulses with durations of approximately 12 ms, producing good quality in vivo inner volume 3D‐FSE T_2_‐weighted brain images.

## THEORY

### Key Features of FSE Sequences

To understand the methods proposed in this work, some key features of FSE sequences will be restated here. First is the property mentioned in the introduction, that assuming sufficient spoiling of FIDs, magnetization not tipped by the excitation pulse cannot yield signal later even if nonselective refocusing pulses are used. Second, 3D‐FSE typically uses sweeps with variable refocusing flip angles, often ending on low values. The reason is partly for reducing specific absorption rate (SAR), however the resulting mix of stimulated and spin echoes also reduces the apparent T_2_ decay allowing more echoes to be acquired per shot [see for example Mugler 6]—essential for 3D imaging which requires a large amount of phase encoding. A great many strategies exist for designing flip angle sweeps; in this work we use the simple approach of pseudo‐steady‐state 11 echo trains consisting of a short sweep of angles, converging to a constant asymptotic flip angle that we use to characterize the train [see for example Figure 2 in Hennig et al 12]. Third, the pulses must satisfy the Carr‐Purcell‐Meiboom‐Gill (CPMG) condition 13, that there is a 90° phase difference between excitation and refocusing pulses, i.e., excitation and refocusing pulses must rotate the magnetization vector around orthogonal axes. If this condition is not met, echo amplitudes rapidly decrease when the refocusing angle is not precisely 180° 13.

### CPMG‐Specific Pulse Design Method

Following the spatial domain small tip approximation (STA) design method 14, the pulse design problem can be written **m** = **Ab** where vector **b** contains the desired radiofrequency (RF) pulse samples, system matrix **A** contains the spatial transmit sensitivities and phase factors related to gradient encoding and **m** is the target flip angle. In this context, **m** is complex, and its phase corresponds to the axis that the magnetization is rotated into, rather than the axis about which it is rotated. Specifying a real valued **m** is equivalent to solving for a flip angle target that has zero phase. This constraint on the design problem can be overly restrictive in many circumstances, for example if the designed pulses are to be used in gradient echo sequences. In this case, it has been demonstrated that by solving only for the magnitude of the excitation magnitude least squares (MLS) methods can result in improved performance 15. Spin echo sequences on the other hand are phase sensitive, because the CPMG condition must be observed. The component of the excited magnetization that is *perpendicular* to the rotation axis of the refocusing pulses (i.e., the non‐CPMG component) will yield echoes that quickly die away through the imaging sequence. If long echo trains are used with the center of k‐space encoded later in echo train (as is the case with linear phase encode ordering) the resulting signals are strongly suppressed. We propose to use this natural suppression mechanism as an additional degree of freedom for pulse design, by trying to push residual errors in the excitation profile to the non‐CPMG component where they will be naturally suppressed. The proposed method achieves this objective by performing a weighted least squares minimization.

The classic optimization scheme proposed by Grissom et al 14 uses scalar weighting factors for each position in space. A straightforward way to implement this is to solve the modified problem:

(1) m ○ w = (A ○ W) b

where the column vector **w** contains a weighting value for each spatial location, the matrix **W** has the same size as system matrix **A** with each column being a copy of **w** (i.e., **W_ij_** = **w_i_**), and ‘ ○’ signifies an element‐wise product. To separately weight the CPMG and non‐CPMG parts of the excited magnetization, we must separate them. Assuming without loss of generality that the refocusing pulses rotate the magnetization about the (rotating frame) y‐axis, then the excited magnetization is required to be aligned with y. Therefore, in a complex representation the target **m** will be pure imaginary, and the real part represents the non‐CPMG component aligned with x. For further clarification of the geometry, please see Supporting Figure S1, which is available online. The design problem may be rewritten to separate real and imaginary components:

(2) $$\left[\begin{array}{l} Im(m\text{)}\\ Re(m) \end{array}\right]=\left[\begin{array}{l} Im(A\text{) }Re(A\text{)}\\ Re(A\text{) }-Im(A\text{)} \end{array}\right]\left[\begin{array}{l} Re(b\text{)}\\ Im(b\text{)} \end{array}\right]$$

which can be expressed as a larger matrix problem

(3) $$m_{s}=A_{S}b_{s}$$

where subscript s indicates the real and imaginary parts are separated. Equation (3) may be solved by performing weighted minimization as in Eq. (1) except that different weighting factors can be introduced for the real and imaginary components:

(4) $$\left[\begin{array}{l} Im(m{\text{) ○ }w}_{i}\\ Re(m{\text{) ○ }w}_{r} \end{array}\right]=\left[\begin{array}{l} Im({A\text{) ○ }w}_{i}{\;Re(A\text{) ○ }w}_{i}\\ {R\mathrm{e(}A\text{) ○ }w}_{r}{\text{ −}Im(A\text{) ○ }w}_{r} \end{array}\right]\left[\begin{array}{l} Re(b\text{)}\\ Im(b\text{)} \end{array}\right]$$

where the vector **w_i_** contains weightings for the imaginary part, **w_r_** contains weightings for the real part and matrices **W_i_** and **W_r_** are concatenated copies of the respective column vectors. Equation (4) is expressed compactly as:

(5) $$m_{sw}=A_{sw}b_{s}$$

and a least squares solution with additional regularization term for RF power can be found by performing the minimization:

(6) $$\underset{b_{s}}{\text{min}}\left\{||A_{sw}b_{s}-m_{sw}|{|}^{2}+\lambda ||b_{s}|{|}^{2}\right\}$$

where λ is an adjustable regularization parameter. In this work, **w_r_** and **w_i_** are treated as spatially invariant, but we explore the use of lower weights for **w_r_** within the range:

(7) $$w_{i}=1.0\;\;w_{r}=[0,1].$$

Setting **w_r_** = 1.0 gives equal weight to both components, and makes the minimization in Eq. (6) equivalent to the standard procedure; reducing **w_r_** results in an optimization weighted toward reducing errors in the CPMG component at the expense of increased errors in the non‐CPMG component. We hypothesize that choosing a low value for **w_r_** could, therefore, result in better performance in the CPMG part of the excitation, with errors pushed to the non‐CPMG part, which will be suppressed.

RF pulses with target flip angles of 90° are designed using the STA; the method can be applied with any target flip angle, however accuracy may fall at high tip angles. Bloch simulations are then used to assess performance by computing an equivalent flip angle α(**r**) from the magnetization vectors 16:

(8) $$\alpha (r{\;)=\text{cos}}^{-1}\left(\frac{M_{z}(r\text{)}}{M_{0}}\right)\text{exp}\left\{i∠M_{xy}(r)\right\}.$$

This quantity is directly comparable with **m**.

The proposed method achieves enhanced background suppression by making residual excitation in outer volume regions violate the CPMG condition. For inner and outer volume locations **r_in_** and **r_out_**, the overall background suppression factors may be quantified as:

(9) $$\begin{array}{l} \sum_{non-CPMG}=\frac{\text{sin}(Im(\alpha (r_{in})))}{\text{sin}(Re(\alpha (r_{out})))}\times \Xi \\ \sum_{CPMG}=\frac{\text{sin}(Im(\alpha (r_{in})))}{\text{sin}(Im(\alpha (r_{out})))}. \end{array}$$

Σ_non‐CPMG_ and Σ_CPMG_ are background suppression factors for non‐CPMG/CPMG outer volume excitations, compared with CPMG inner volume excitation with flip angle Im(α(**r_in_**)). Σ_CPMG_ simply depends on the sine of the flip angles of the inner and outer volume regions—this is the typical form of background suppression. Σ_non‐CPMG_ has an additional “non‐CPMG suppression factor” Ξ defined as the ratio between echo amplitudes from CPMG and non‐CPMG excitations with the same flip angle . Ξ is tissue and sequence specific and can be estimated by means of extended phase graph (EPG) simulations as described in the methods section; the value of Ξ represents the additional background suppression available from the proposed method (Ξ≥1).

## METHODS

### Overview of Pulse Design Method

Inner volume excitation requires specification of both gradient and RF waveforms. Optimal k‐space (and hence gradient) selection is an unsolved issue for multidimensional pulse design; this work used a fixed “3D shells” k‐space trajectory 3 for all subjects, with RF pulses designed on a per‐subject basis. The 3D shells approach has the advantage of sampling the k‐space center at the end, making the resulting pulses suitable for spin echo imaging where the time between the effective center of the excitation pulse and the first refocusing pulse determines the echo spacing that may be used. Time‐optimal gradient waveforms respecting maximum slew rate 200 T m^‐1^s^‐1^ and gradient strength 40 mT m^‐1^ were produced using the method of Lustig et al 17 with code obtained from the author's website. Corresponding RF pulse waveforms were obtained using the proposed phase relaxed design method, for a target excitation flip angle of 90°. Simple minimization as outlined in Eq. (6) can lead to pulse amplitudes that violate peak forward power constraints; the iterative “re‐VERSE” method 18 was used to reduce peak amplitudes. Impulse response functions were used within the re‐VERSE and time‐optimal gradient design methods to account for nonideal performance of the gradient system.

### Compensation for Nonideal Gradients

Nonideal gradient performance affects the relationship between the nominal gradient waveforms and achieved k‐space trajectory. This is also an issue for the time‐optimal gradient method 17, and the implementation of re‐VERSE 18 which is based on it, because the hardware constraints (maximum amplitude and maximum slew rate) apply to the nominal gradient waveform, whereas the k‐space trajectory depends on the achieved gradient fields. Some pulse design methods have been proposed that use in situ gradient measurements to update the designs 19, 20. The requisite measurements can be obtained by means of imaging [for example, Duyn et al and Papadakis et al 21, 22] or dedicated probes [for example De Zanche et al 23] if available. As an alternative to physical measurement of achieved gradient fields, it has been shown that the gradient system may be approximated as a linear time invariant (LTI) system characterized by an impulse response function **H**(t) 24, 25. In this work, we use such an approach. Gradient waveforms **G**(t) are designed using the time‐optimal method for an input k‐space profile, then RF pulses are designed for the predicted k‐space trajectory **k_H_**:

(10) $$k_{H}\{\text{G(}t)\}=-\gamma \int_{t}^{T}\{H*G\}(\tau )d\tau .$$

If the maximum RF amplitude limit is exceeded, then the time‐optimal VERSE 26 method is used to produce an updated gradient waveform, which is used as the input for the next iteration of the algorithm. Impulse response functions for each gradient axis were measured using chirped input pulses; gradient fields were measured using an image based method similar to that described in Papadakis et al 22. The measured impulse response function is shown in Supporting Figure S2.

### Trajectory Selection

The k‐space trajectory used in this study was fixed a‐priori for all subjects based on investigations on test data. Whole head B_1_ and B_0_ maps (protocols described later) were used to evaluate the point spread function (PSF) of candidate k‐space trajectories using the method outlined by Schneider et al 3, which considers the result of designing a pulse for a point‐like target as a surrogate for the PSF (though the PSF is spatially variable, it is evaluated at a single location and results should be treated as a guide for general performance). For each candidate trajectory, k‐space curves were defined using the method of Wong and Roos 27; time optimal gradient waveforms were generated for these, then **k_H_** was obtained. Pulses were designed to excite a single voxel on the design mesh, then the forward model was used to predict the resulting excitation. A set of 2000 candidate trajectories was generated by randomly varying the number of shells, sampling density, maximum radial k‐space extent, and angular offset between shells [this is not present in Schneider et al 3]. The final choice of trajectory was made by considering properties of the PSF (full width half max and peak to sidelobe ratio) as well as duration. The choice was made manually rather than by means of specific numerical criteria. Although the pulse duration does not directly limit the speed of the rest of the imaging sequence, durations below 15 ms were targeted to minimize T_2_^*^ effects.

### EPG Simulation Study of Background Suppression

EPG simulations were used to assess suppression of non‐CMPG excitation (i.e., Ξ from Eq. (9)). Refocusing angle sweeps can be generated by multiple methods, in this work we investigated simple pseudo‐steady state 11 sweeps, computed using the “one‐ahead” algorithm as detailed in Hennig et al 12. Each set of refocusing angles consists of a sweep down to a constant asymptotic angle, which we use as a label. For example, the sequence used for all imaging experiments had asymptotic refocusing angle 35° (refocusing angles 139°, 74°, 50°, 41°, then constant 35°). Note that this refocusing sweep was selected because it is appropriate for T_2_‐weighted brain imaging, and is used in a product T_2_‐weighted 3D‐FSE sequence on our scanner.

Echo amplitudes were computed for CPMG and non‐CPMG excitations of 90°, in conjunction with 100 echo FSE sequences with interecho spacing 4 ms. Three brain compartments were simulated: gray matter, T_1_ = 1543 ms, T_2_ = 122 ms; white matter, T_1_ = 907 ms, T_2_ = 92 ms; and cerebrospinal fluid (CSF), T_1_ = 3651 ms, T_2_ = 1429 ms 28. Assuming the central echoes in the train dominate the image signal (i.e., linear phase encoding), we calculate Ξ as the ratio of the average echo amplitudes for the 10 central echoes (i.e., echoes 46 to 55).

### Experiments

All experiments were conducted using a 3 Tesla (T) Philips (Best, Netherlands) Achieva MRI scanner fitted with an eight‐element parallel transmit body coil, described in Vernickel et al 29. An eight‐channel head coil was used for signal reception. For in vivo experiments, SAR was estimated using an electromagnetic model of the transmit coil simulated using CST Microwave Studio 2013, with the NORMAN voxel model 30 positioned with head at isocenter. The numerical model was tuned and matched as described in Beqiri et al 31, and local SAR was evaluated using the model compression approach outlined in Eichfelder and Gebhardt 32 with 1% overestimate bound.

A phantom experiment was performed to explore performance of the proposed phase relaxed method with variable **w_r_**. For simplicity, 2D selective pulses were designed with a 2D spiral k‐space trajectory, and these were imaged using a single shot 2D‐FSE sequence with long echo train and slice selective refocusing pulses; the proposed CPMG signal suppression mechanism applies equally to 2D and 3D imaging, but 2D images are simpler to evaluate. These designs were not combined with the described re‐VERSE method, instead achievable maximum pulse amplitudes were obtained by using a variable velocity spiral trajectory similar to that described in Xu et al 33. An arbitrary inner‐volume (a heart shape of maximum width 55 mm) was defined and apodized with a Gaussian kernel of size 3 mm. The target was offset diagonally from isocenter to avoid overly favorable performance with the spiral trajectory. Ninety‐degree excitation pulses were designed using Eq. (6) for a 2D spiral trajectory with nominal resolution 3.4 mm and under‐sample factor 3.5 (duration 10.0 ms)—the predicted k‐space was used in the design. The multishift conjugate gradient method 34 was used to solve Eq. (6) for multiple λ simultaneously. All pulses were designed using MATLAB R2014 (The Mathworks, Natick, MA) running on a Dell Precision T5600 Workstation (Round Rock, TX) with 12 cores and 64GB of RAM.

The phantom was a spherical flask filled with a solution of CuSO_4_ with measured T_1_ = 270 ms and T_2_ = 220 ms. The single shot 2D‐FSE sequence had field of view (FOV) 220 mm × 220 mm, resolution 2 mm, slice thickness 2 mm, echo train length 116 (including 6 dummy echoes), echo spacing 4.1 ms and asymptotic refocusing flip angle 35° as described above. Linear ordered phase encoding was used. Ten signal averages were obtained with repetition time 5 s. The 3D spoiled gradient echo (3D‐SPGR) images were acquired using the same excitation pulses scaled down to produce flip angle 10°, at the same in‐plane resolution with 5 mm slices and repetition time 20 ms.

The 3D‐FSE IVI was performed on four adult volunteers and on the phantom described above; ethical approval was obtained for the study and all subjects gave written consent. In each case, a 90° excitation was designed for a 60‐mm cube target (apodized with 6‐mm kernel) placed approximately within the cerebellum. To manage memory requirements, 3D pulses were designed on grids with 5.5‐mm resolution. The iterative re‐VERSE method was used as described above with a maximum of five iterations; a maximum pulse amplitude of 11 μT was targeted by limiting the amplitude to 8 μT at each time‐optimal VERSE iteration; this so‐called “attenuation factor” is described in Lee et al 18. Weighting factor w_r_ = 0.25 and regularization parameter λ = 100 were used for all 3D designs; these were selected based on pilot data performance. Computation time was approximately 25 s per iteration, giving overall time of under 3 min, which is acceptable for on‐line design.

Two separate 3D‐FSE imaging sequences were used, one encoding the whole head and the other encoding just the inner volume region; both with frequency encoding head‐foot. Both sequences used a train of 106 echoes (including 6 initial dummy echoes) using asymptotic refocusing angle 35° and linear ordered phase encoding. The repetition time was 2.5 s and the echo spacing was 4.0 ms, all refocusing pulses were nonselective. A fat‐saturation pulse was used before excitation; this is crucial because the localized excitation pulses have rather narrow bandwidth and were designed only for the water frequency. The large FOV sequence had 1 mm isotropic resolution with SENSE factor 1.7 in both phase encode directions (i.e., factor 3.4 under‐sampling) and total imaging time 5 min 0 s; the small FOV sequence had 0.8 mm isotropic resolution with no parallel imaging acceleration and total acquisition time 3 min 17 s. For the phantom experiment, 3D‐SPGR images were also acquired for evaluation purposes using the same FOV and resolution as the large FOV 3D‐FSE scan, with repetition time 30 ms.

The 3D B_0_ and B_1_ field maps were required for pulse design; these were acquired for all experiments using the same protocols. B_1_ maps were acquired using a combination of a single quantitative map in quadrature mode and per‐channel relative maps 35. Quantitative maps were acquired using the actual flip angle imaging method (AFI) 36 with flip angle 80° and repetition times 30 ms and 150 ms. Relative sensitivity maps were acquired for linear combinations of channels 37, 38 using 3D gradient echo imaging with flip angle 1° and repetition time 3.5 ms. Separate 3D B_0_ field maps were acquired with a dual gradient echo sequence with water and fat in‐phase (2.3 ms, 4.6 ms). All maps were acquired at 5 mm isotropic resolution.

### Quantitative Suppression Measurements

Suppression was measured by comparing the average signal inside a 1 cm^3^ volume within the excited inner volume with measures of the signal in the outer volume region. A 60 mm FOV was excited, but zoomed imaging used an 80 mm encoded FOV; hence, only signal outside a 100 mm cubic volume could alias into the 60 mm FOV. The outer volume was defined as all regions outside the 100 mm cube but still within the imaged object. In vivo scans had a large range of signal values, dominated by high signal from CSF; for these scans the small volume used for quantifying average signal was placed over a region containing CSF, although it also contained signal from other tissues. For the phantom scans the outer volume region was eroded slightly to exclude the very edge, which was the source of an unrelated image artifact (described later).

### Code Availability

Source code for 2D and 3D pulse design along with example binary data can be downloaded from https://github.com/mriphysics/phase_relaxed_CPMG_excitation. A citeable version with persistent identifier doi:10.5281/zenodo.19957 links to release 1.0 of the code, current at time of publication.

## RESULTS

**Figure 1:**
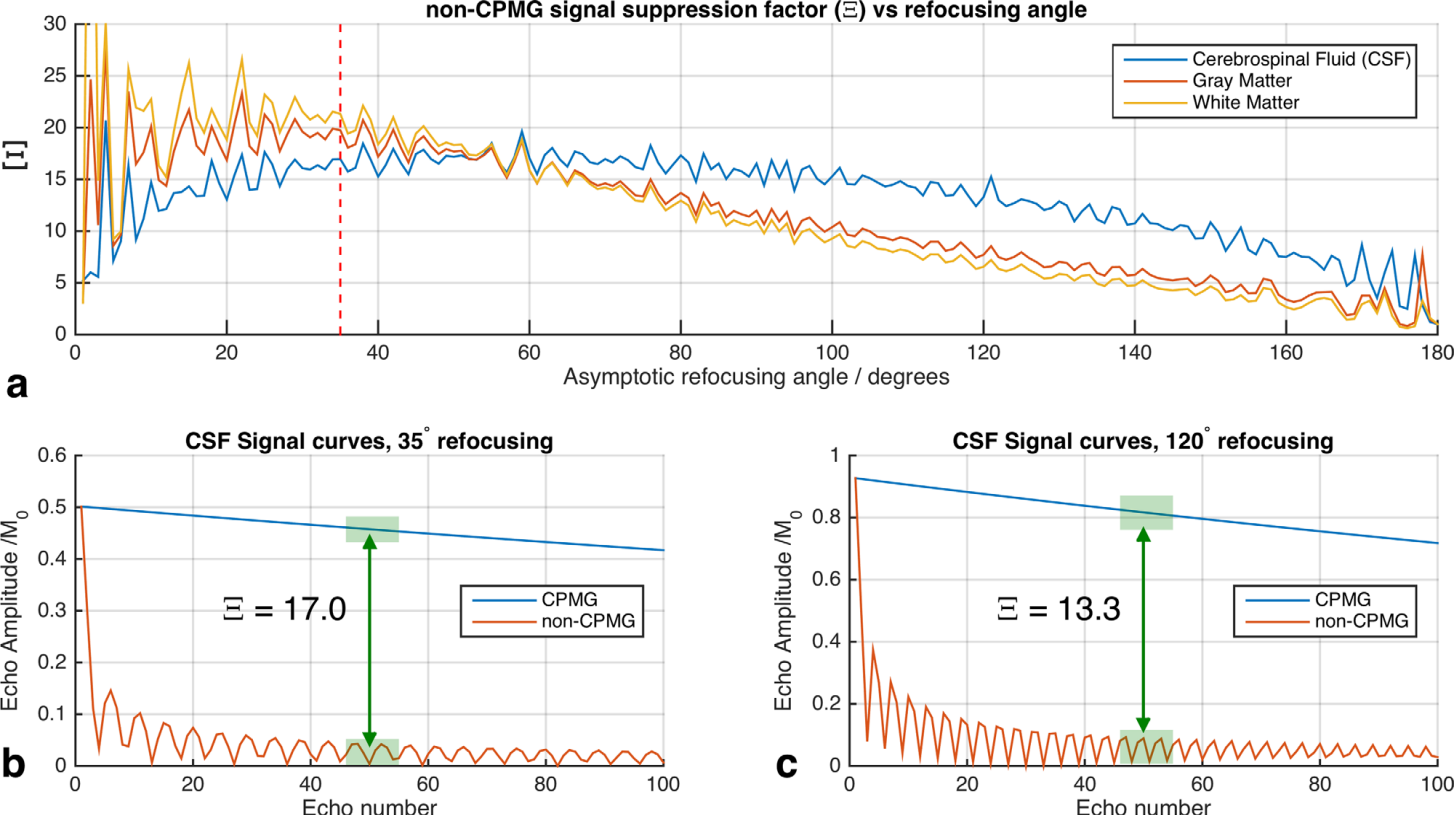
Simulation study of non‐CPMG signal suppression factor Ξ for 100 echo FSE sequences with differing refocusing flip angles (35° was used for imaging in this work). Ξ is calculated as the ratio of the mean signal from CPMG and non‐CPMG excitations with the same excitation flip angle (i.e., same absolute flip angle but 90° phase difference between the two cases). “Signal” is here estimated as the mean echo amplitudes for the 10 echoes at the center of the echo train (i.e., linear phase encode ordering is assumed). **a:** Ξ versus refocusing angle for three tissue types. **b,c:** Example echo trains CSF for refocusing angle 35° (b) and 120° (c) both in CSF. Ξ is indicated by annotation on parts (b) and (c). Note that (b) and (c) compare CPMG and non‐CPMG signals from a 90° excitation. As described by Eq. (9), non‐CPMG suppression is only part of the available background suppression, which also relies on creating excitations with small flip angles in the outer volume.

Figure 1 summarizes predicted non‐CPMG suppression factor Ξ for various sequences with different refocusing flip angles. Example echo amplitudes are depicted on Figures 1b and c; the annotations show examples of how Ξ is computed. Figure 1 indicates that the 35° refocusing sequence used in this work coincides with a high degree of available suppression, with Ξ≈20. However, Figure 1 also shows that higher refocusing angles of 120° can still yield Ξ>10 for CSF.

The example echo amplitudes in Figures 1b and c show that the non‐CPMG signal declines quickly. Following Eq. (9), the overall background suppression factor depends on the excitation flip angle as well as Ξ. For example a sequence with Ξ = 17 (CSF suppression factor from Figure 1) used with a perfect inner volume excitation of Im(α(**r_in_**)) = 90° with some residual non‐CPMG outer volume excitation of Re(α(**r_out_**)) = 10° results in overall background suppression factor 98.

### 2D Experiments

**Figure 2:**
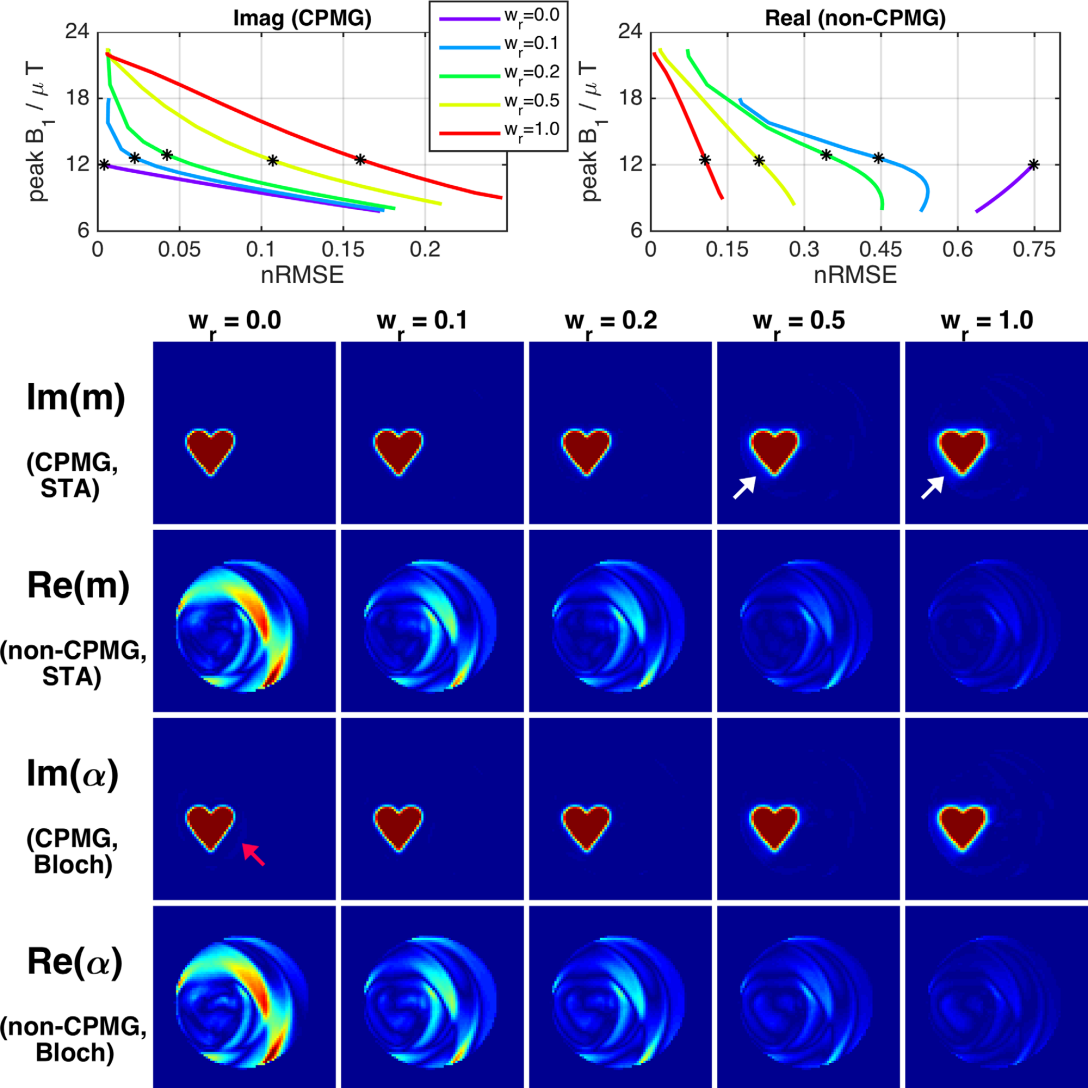
Results of phase relaxed optimization for 2D spiral experiments. Errors in Im(**m**) (the CPMG component) and Re(**m**) (non‐CPMG) are plotted against peak pulse amplitude; the solutions marked by asterisks are displayed in the images below. The images display the solutions as predicted by the STA (linear model) (**m**) as well as the results from Bloch simulation (**α**, as defined in Eq. (8)). All images use a color scale ranging from 0° to 60°. For w_r_ = 0 there is a large error in Re(**m**) (which should be zero) but very high fidelity in Im(**m**). As w_r_ is increased the error in Re(**m**) falls at the expense of Im(**m**). Unsuppressed background excitation is present for the two largest values of w_r_, as emphasized by the white arrows. Bloch simulation results are very similar to the linear model, however some differences are apparent (e.g., unsuppressed background in Im(**α**) for smallest w_r_, red arrow).

**Figure 3:**
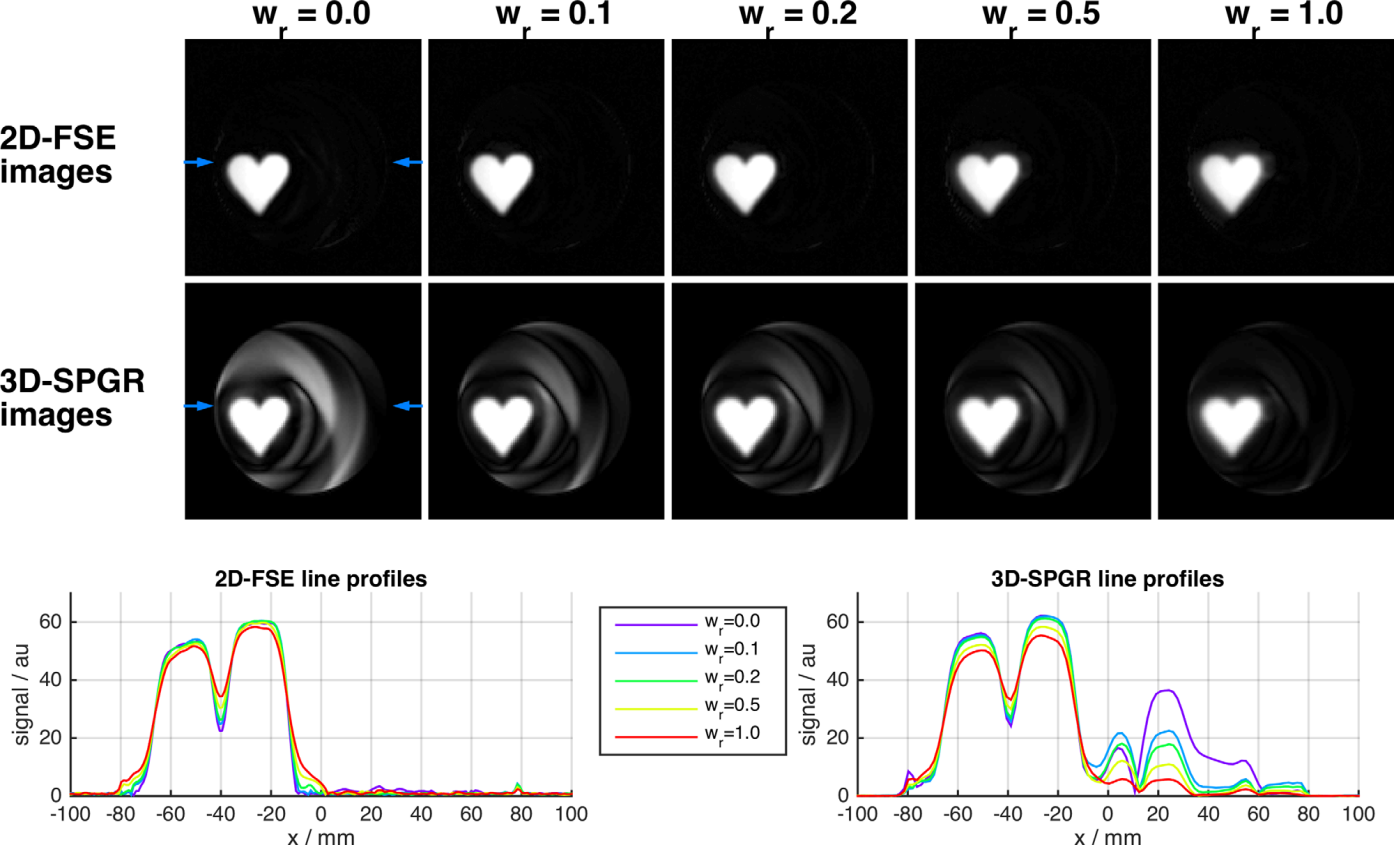
Imaging results from 2D spiral experiment (equivalent central slice from 3D‐SPGR sequence is shown; the 2D‐FSE used slice selective refocusing). Comparing with Figure 2, the FSE images closely resemble Im(**α**) (CPMG) indicating that Re(**α**) (non‐CPMG) has been largely suppressed by the imaging sequence. Line profiles were taken at the level indicated by the blue arrows. Profiles from the 2D‐FSE images indicate that the excitation becomes increasingly blurred as w_r_ is increased from zero. The very poor background suppression in the SPGR images is expected because this sequence is sensitive to |**α**|; the SPGR images closely resemble the predicted magnetization in Figure 2.

Figure 2 shows error in Im(**m**) and Re(**m**) (CPMG and non‐CPMG) versus peak RF amplitude for solutions obtained with different λ and **w_r_**. Each individual curve plots solutions for multiple values of λ at fixed w_r_; solutions with maximum peak amplitude within system constraints were selected for imaging (asterisks). The curves show that as w_r_ is reduced from 1.0 the error in the CPMG part of the excitation (Im(**m**)) falls at each peak amplitude level, and the error in the non‐CPMG part (Re(**m**)) increases; the relative change is however different. As w_r_→0 errors in Re(**m**) grow quickly with diminishing reductions in error in Im(**m**). This is also apparent in the images, which display predictions from the linear model (**m**) along with results from Bloch simulation (**α**). For this design problem, the normal method of w_r_ = 1.0 results in a poor solution for Im(**m**) with a spatially blurred profile and some non‐zero excitation outside the target area (white arrows). The Bloch simulations show that the excitation pulses perform well at 90°; however, Im(**α**) does deviate subtly from Im(**m**), with some unsuppressed background in the former (red arrow). The imaging results in Figure 3 compare 2D‐FSE with 3D‐SPGR images; 3D encoding is necessary for the SPGR because it lacks the slice selective refocusing pulses used to limit signal to one slice with the FSE. Figure 3 demonstrates the central premise to the proposed phase relaxation method; the 2D‐FSE images contain a very low level of signal outside the intended excited region even in the case where the non‐CPMG part of the excitation is large (compare with Figure 2). This is in striking contrast with the 3D‐SPGR images which are influenced by |α| and contain large amounts of signal outside the local volume, as expected. Line profiles of the 2D‐FSEs indicate that the definition of the local volume becomes successively more blurred as w_r_ is increased, which is in agreement with the increasing errors in Im(**m**) seen on the L‐curves in Figure 2. There is a small amount of residual signal in the background for the w_r_ = 0 image where Re(**α**) was particularly large (see line profile at x = 20–30 mm).

### 3D Selective Excitations

**Figure 4:**
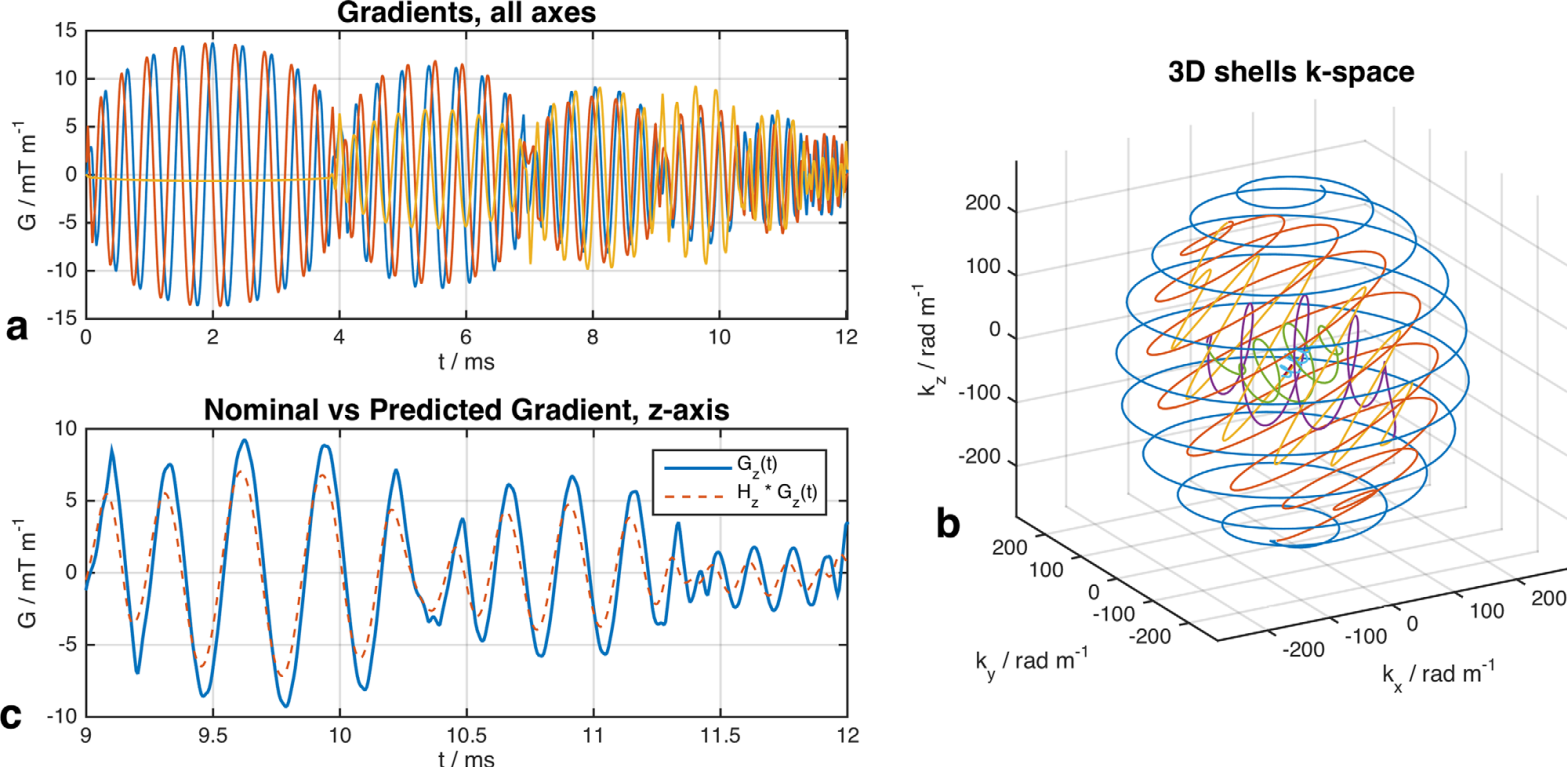
Gradient waveforms (**a**) and predicted k‐space **k_H_**(**G**(t)) trajectory (**b**) selected for experiments. The individual “shells” in the k‐space diagram are colored differently for clearer visualization; in this trajectory successive shells are offset by 30° rotation around the x‐axis. **c:** Comparison between nominal (**G**(t)) and predicted ({H*G}(t)) waveforms for a 3 ms portion of the z‐axis gradient.

The 3D shells trajectory used in this work was selected to yield relatively small full width half maximum (FWHM) (21 mm) and high peak‐to‐sidelobe ratio 32. The trajectory had six shells, maximum radius 280 rad m^−1^, 30° offset between shells, radial under‐sample factor 1.75, angular under‐sample factor 3.21, and total duration 12.02 ms (under‐sample factors relative to 220 mm FOV). This is illustrated in Figure 4 alongside differences between nominal and predicted gradient waveforms; the latter are used to compute k‐space for all calculations (see Supporting Figure S3 for k‐space comparison). More detail on the properties of the randomly generated candidate trajectories is given in Supporting Figure S4 and Supporting Table S1.

**Figure 5:**
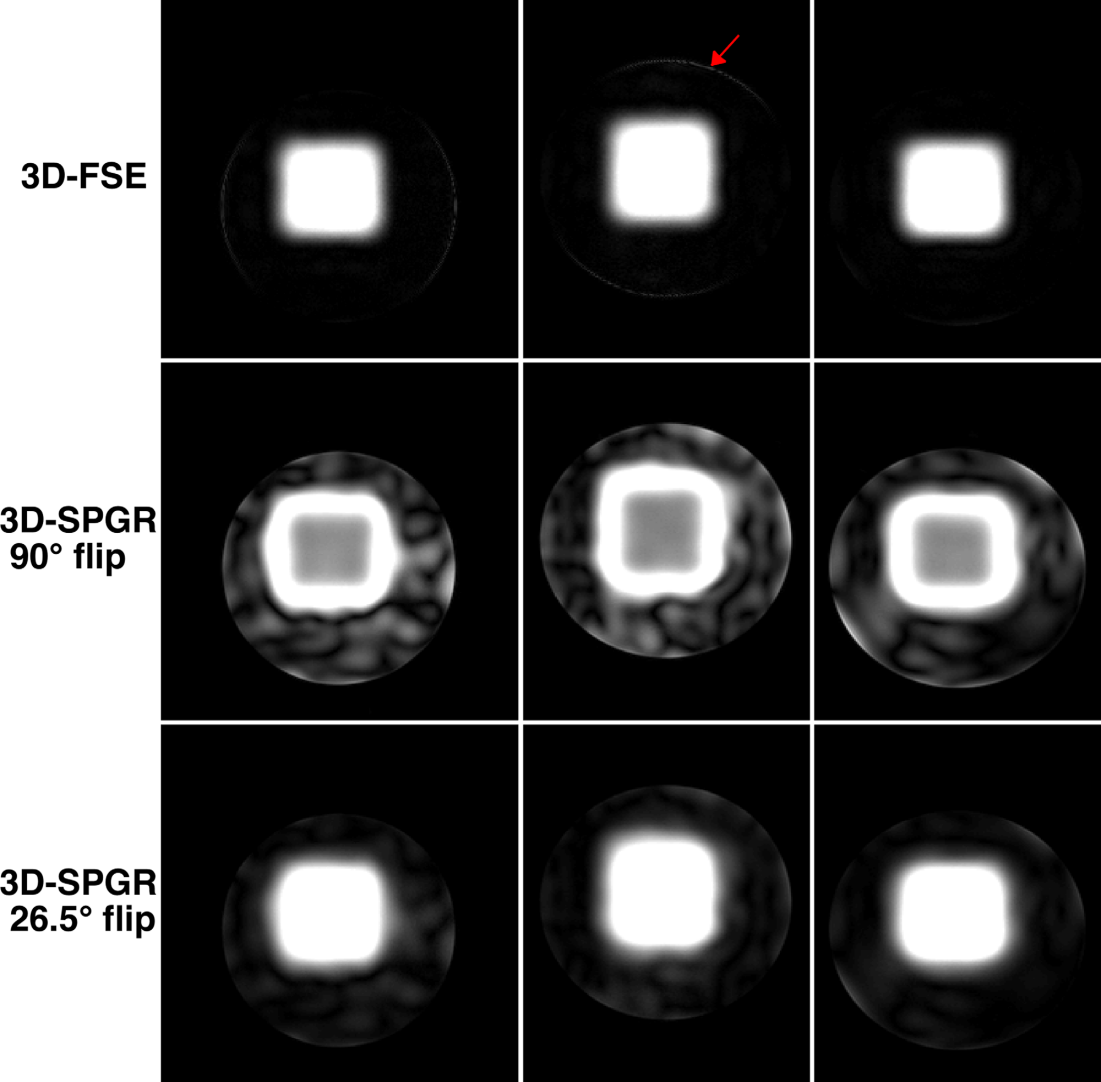
Imaging results for 3D‐shells excitation in phantom (the columns represent three orthogonal views). Images shown with same relative windowing. The 3D‐SPGR with the 90° excitation shows significant background signal and a high degree of saturation in the inner volume, while 3D‐FSE shows low background signal. The 3D‐SPGR was also acquired with the RF pulse scaled to the Ernst angle for this sequence (26.5°). This compares with the 3D‐FSE but a high degree of unsuppressed background signal is visible. An “edge” artifact present in the 3D‐FSE is highlighted by an arrow; this is interpreted as an FID artifact, discussed in the text.

Figure 5 shows the resulting 3D excitation in a phantom, imaged using 3D‐FSE and 3D‐SPGR and displayed in three orthogonal planes. As with the 2D results, the 3D‐FSE images show good background suppression and a cube excitation is clearly achieved. Two example SPGR images are included; using the intended 90° flip angle and also scaling the pulses down to the Ernst angle to avoid saturation effects. Background suppression in the 3D‐FSE is clearly better than in the 3D‐SPGR with 26.5° flip.

**Figure 6:**
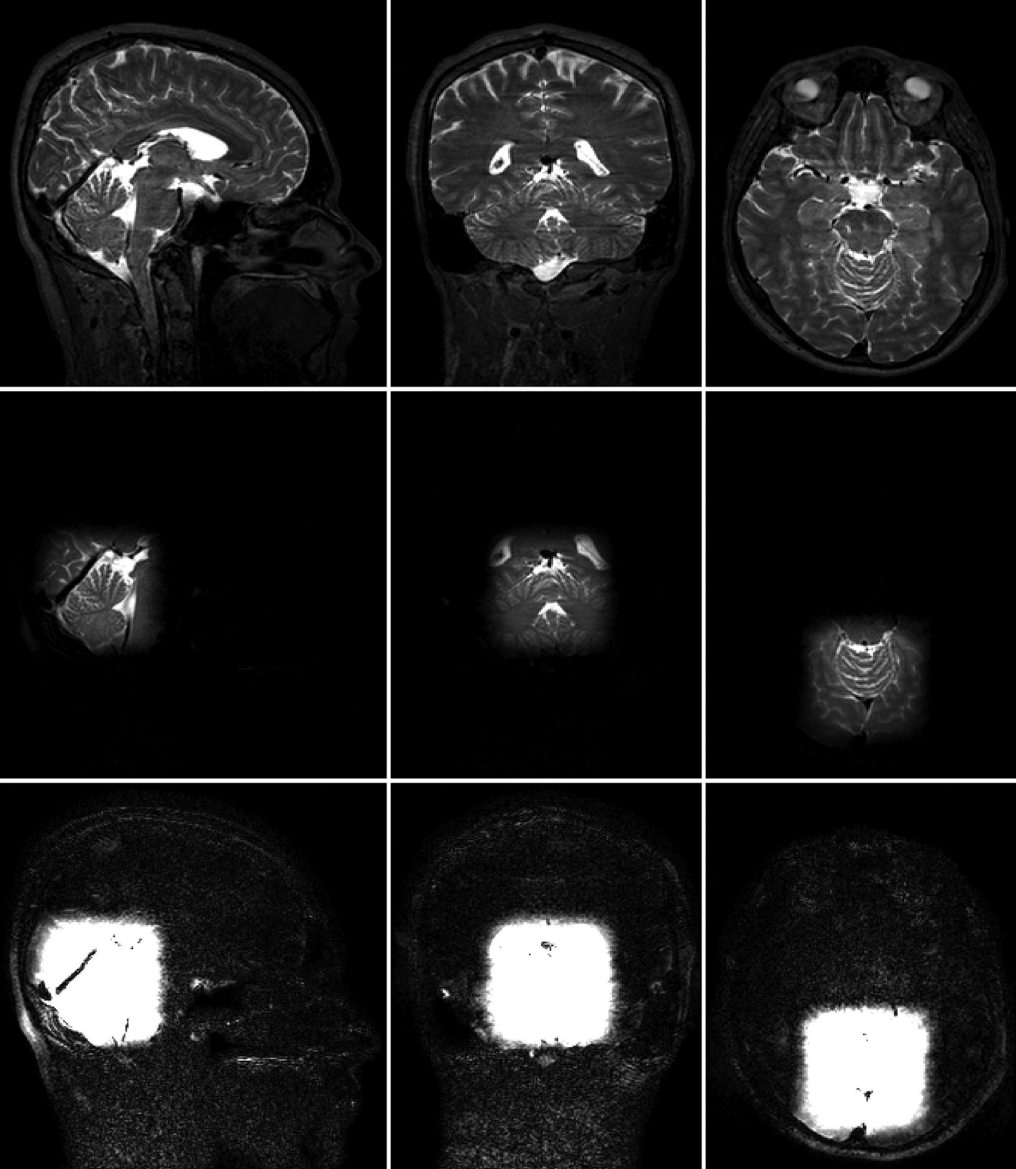
In vivo full field of view 3D‐FSE results; columns represent sagittal, coronal, axial views. Top row: 3D‐FSE with standard hard pulse excitation. Middle row: 3D‐FSE with 3D‐shells excitation pulse. Bottom row: 3D‐shells image with colormap scaled by factor 8 to show background signal. Good background suppression is apparent; noise appears to be confined to the head because the background outside the head is masked by the scanner SENSE reconstruction. Small highly focal regions of out of volume signal can be seen close to ear canals and nasal cavity. Some of the images in this figure were published in Padormo et al 47; reproduced with permission.

**Figure 7:**
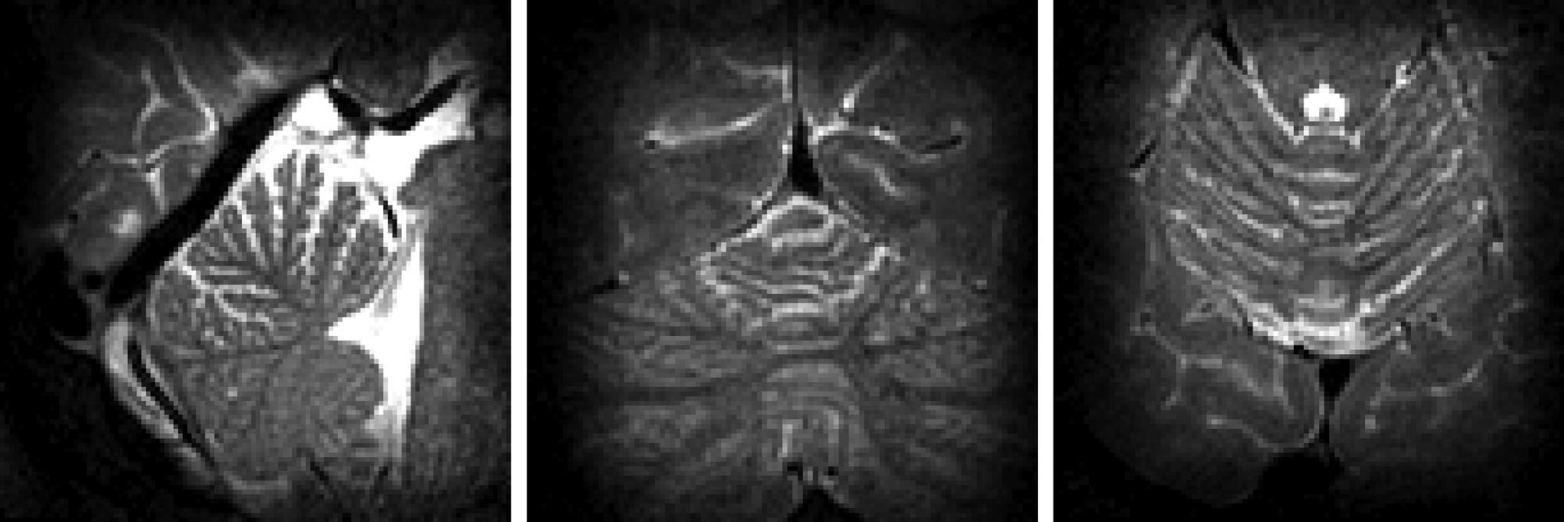
In vivo reduced field of view 3D‐FSE images acquired with 0.8 mm isotropic resolution in 3 min 17 s. *Some of the images in this figure were published in Padormo et al* 47; *reproduced with permission*.

Figure 6 shows a resulting 3D excitation from an in vivo 3D‐FSE scan compared with the standard nonselective excitation. As with the phantom experiments, the background is highly suppressed, and is only visible on the intensity windowed images (bottom row). Note that the background noise appears limited to the head; this is caused by the scanner's SENSE reconstruction, which masks background regions from the coil sensitivity maps. Figure 7 shows the small FOV 3D‐FSE image from the same subject; a high resolution has been achieved with no obvious artifacts. The ratio of inner volume signal in 1 cm^3^ of tissue to mean outer volume signal was 103 for the phantom and 82 ± 6 for in vivo experiments. The ratio of the same inner volume signal to 99^th^ centile outer volume signal was 33 for the phantom and 20 ± 3 in vivo.

**Figure 8:**
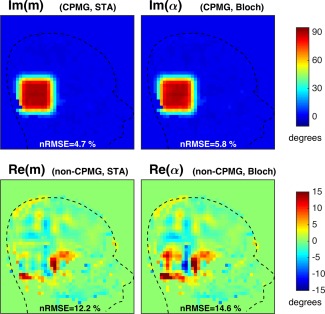
Example RF design for in vivo subject. Images show predicted performance from linear model (m) and full Bloch simulation (α).

**Figure 9:**
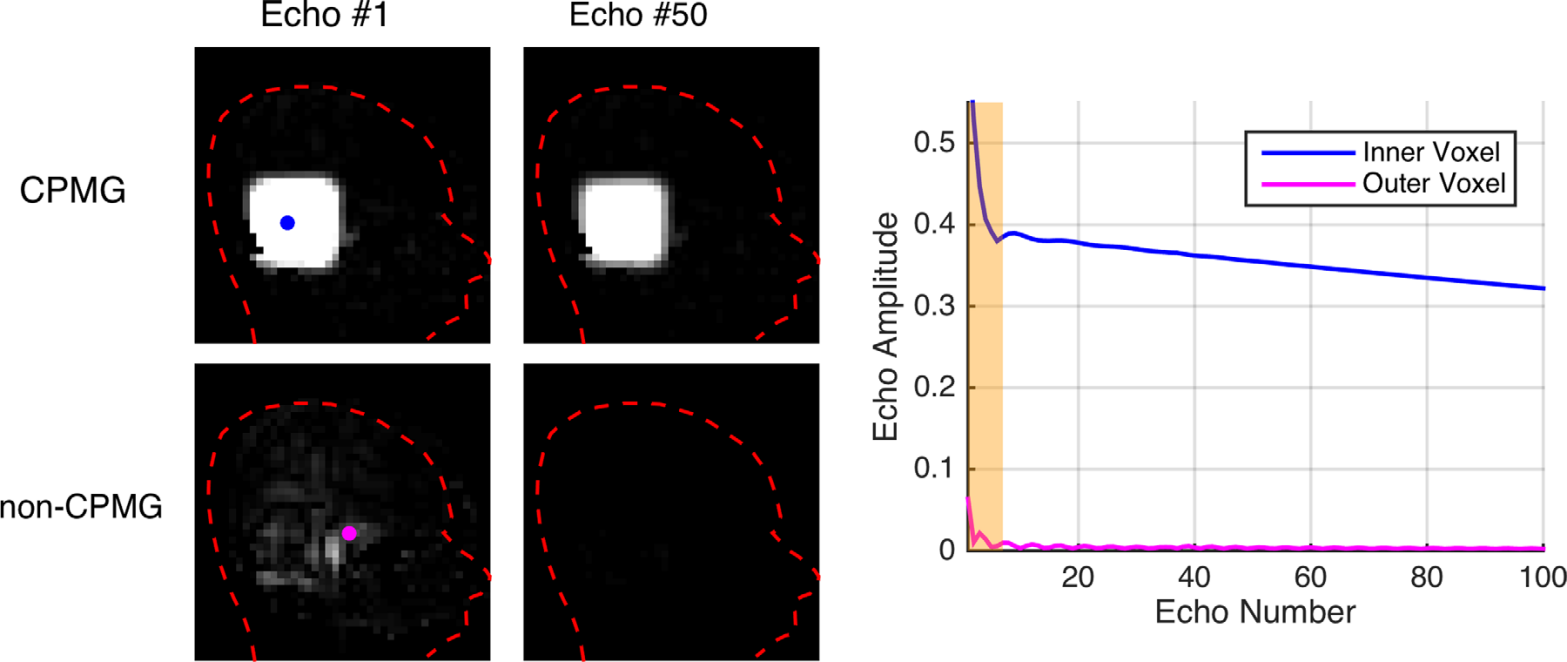
Spatially Resolve EPG simulated echo amplitudes for the predicted excitation in Figure 8. Echo amplitudes have been separately predicted for the CPMG (Im(α)) and non‐CPMG (Re(α)) part of the excitation. The non‐CPMG parts decay quickly, as can be seen in the plots (shaded region corresponds to dummy echoes not used for imaging encoding). Note that these simulations treat the whole head as having the tissue properties of CSF; signals from other brain tissues decay much more quickly.

An example in vivo designed excitation is given in Figure 8. While there is some difference in the overall error level (nRMSE in Im(**m**) increases from 4.7% to 5.8%) the STA design performs well at 90° flip angle. The predicted excitations from Figure 8 were used within a spatially resolved EPG 39 simulation to predict the resulting echo amplitudes as shown in Figure 9. The whole head is treated as having the tissue properties of CSF (T_1_ = 3651 ms T_2_ = 1429 ms) 28; signals from other brain tissues decay much more quickly. The CPMG part of the excitation (Im(**α**)) creates stable echoes within the inner volume region, whereas the non‐CPMG part produces signals in the outer volume that quickly diminish in time. The center of k‐space of the imaging sequence is covered at the center of the echo train, and by this time the outer volume signal has significantly reduced.

An example set of waveforms is given in Supporting Figure S5; the pulse amplitude was successfully limited by using the re‐VERSE approach. Pulse durations of 12.5 ± 0.2 ms were obtained (re‐VERSE alters the pulse duration). SAR for all in vivo optimized pulses is quantified in Table 1. The low refocusing angles mean that this is already a low SAR sequence, with local SAR of approx. 1W kg^‐1^ using standard nonselective pulses. On average the 3D shells pulses produce approximately double the local SAR of the standard hard pulse. However this amounts to only around 10% of the total sequence SAR.

**Table 1:** SAR for 3D Shells Pulses (Averaged over All In Vivo Subjects) Compared to Standard Pulses Used in Quadrature Mode^a^

Pulse(s)	Head average SAR		max. 10g SAR	
	(mW kg^−1^)	Compared to limit (3.2W kg^−1^)	(mW kg^−1^)	Compared to limit (10W kg^−1^)
3D Shells excitation	11.5 ± 1.0	0.36 ± 0.03 %	95.6 ± 8.8	0.96 ± 0.09 %
Hard pulse excitation	6.1	0.20 %	53.6	0.54 %
Rest of sequence	104.1	3.25 %	918.4	9.18 %
Total with hard pulse	110.2	3.44%	972.0	9.72 %
Total with 3D shells	115.6 ± 1.0	3.61 ± 0.03%	1014.0 ± 8.8	10.14 ± 0.09 %

a ‘Rest of sequence’ includes fat saturation and all refocusing pulses; the overall sequence SAR is the sum of this and either the hard pulse or 3D shells pulse depending on which is used. Repetition time 2.5 s was used to compute all values. SAR values are quoted in mW kg^−1^ and also relative to IEC guideline limits (3.2 W kg^−1^ head average and 10 W kg^−1^ maximum local 10 g average).

## DISCUSSION AND CONCLUSIONS

This work proposed a new phase‐relaxed design method for multidimensional spatially selective RF pulses, specifically tailored for FSE sequences with long echo trains. By using a weighted optimization, high quality CPMG compliant excitations can be obtained, at the expense of producing some additional non‐CPMG compliant errors. EPG simulations confirm that echoes resulting from undesired non‐CMPG part of the excitation are suppressed; for long echo trains with low refocusing angles and linear ordered phase encoding (typical of T_2_‐weighted 3D‐FSE imaging) this mechanism can result in approximately 20‐fold additional suppression. In addition to the phase relaxed design method, this work also used time‐optimal gradient waveforms 17 and the iterative re‐VERSE procedure 40 with modifications to account for gradient system imperfections by means of measured impulse response functions.

The method was demonstrated to work for inner volume imaging with true 3D selective excitation pulses for four subjects in vivo with an eight‐channel PTx system. Good image quality was achieved in vivo (Figures 6 and 7) with strong background suppression. To objectively quantify the suppression achieved it is necessary to look at the 3D‐FSE images directly, because the sequence itself contributes to the background suppression by means of the phase relaxed design. In related work on inner volume 3D‐FSE, Mitsouras et al 5 quantified suppression by comparing the mean signal in a small region in the inner volume with the mean signal in a region in the outer volume. Using a similar approach, we measured suppression ratios of approximately 100 in the phantom and 80 in vivo comparing favorably with reported values of around 25 for a 2D spiral excitation in 5. There are some focal areas of unsuppressed signal visible in the windowed images on Figure 6; these were observed in regions of very quickly varying B_0_. These focal unsuppressed background signals represent excited magnetization that did *not* violate the CPMG condition, and was likely caused by a failure of the design algorithm to account for the strong B_0_ variation in these areas. The design method included a B_0_ map, however, a coarse grid (5.5 mm isotropic) was used to reduce memory requirements. The presence of these artifacts indicates this resolution was insufficient in these areas. The suppression ratio with respect to the 99^th^ centile of outer volume signal was measured at around 20 in vivo, indicating potential worst case focal artifact levels of approximately 5%.

The use of low refocusing flip angles in FSE sequences generates FID signals that must be suppressed by suitable spoiler gradients. The bright signal seen at the edges of the phantom images on Figure 5 (red arrow) are an example of this issue. FID artifacts appear as edge effects primarily in the readout direction because they originate from the high k‐space frequencies of the dephased FID [see for example Figure 13 in Mugler 6]. Mugler 6 provides in‐depth analysis of this issue, indicating that short T_1_ times exacerbate the effect, perhaps explaining why these artifacts were seen in the phantom (whose T_1_ was 270 ms) but not in vivo. Mugler also discusses schemes for removing these artifacts: in addition to spoiler gradients, phase cycling can be used to effectively remove them. The product 3D‐FSE sequence used in this work used only gradient spoiling but was sufficiently well tuned to avoid the issue in vivo.

An alternative inner volume selection method is to use orthogonal 1D selective pulses for excitation and refocusing. Although simpler to implement, a disadvantage of using 1D pulses is that all refocusing pulses must be 1D selective, increasing duration and hence interecho spacing compared with hard pulses. A more sophisticated approach proposed by Mitsouras et al 5 is to use a pair of 1D pulses as preparation for a subsequent train of hard refocusing pulses; however, a disadvantage is that echo times must be constrained and additional spoiling used to avoid spurious echoes resulting from transverse magnetization that is created by the excitation but not subsequently refocused 5. Extension to 3D localization would require more sophisticated echo management, and this was not explored by Mitsouras et al.

As Schneider et al observed with their work on 3D excitations in animals 3, quality of inner volume selection is greatly enhanced by keeping pulse durations short. On a technical level, shorter pulses are easier to design (the required memory for computation scales linearly with duration) and are easier to realize (amplifier stability and droop becomes an issue for very long pulses). Short durations also minimize distortion due to T_2_^*^ and ensure that selection bandwidth is not extremely narrow. We achieved durations of 12.5 ± 0.2 ms in vivo by using a highly under‐sampled k‐space trajectory (approximately factor 5.6). This is comparable to Haas et al 4 who also used 3D‐shells for inner volume selection in human brain, with pulse duration 11.6 ms. The achieved degree of background suppression is not clear in Haas et al 4.

The phase relaxed design method that forms the key contribution of this work was examined using simulations and phantom experiments. EPG simulations suggested that for the imaging sequence used in this work (35° refocusing pulses), residual non‐CPMG background excitation of Re(α) = 10° would yield signals that are a factor of 98 lower than the signals from the inner volume excited with Im(α) = 90°. Testing with a simple 2D problem demonstrated the considerable suppression available by this mechanism. The 2D‐FSE and 3D‐SPGR images in Figure 3 are strikingly different, with very large outer volume signals apparent in the gradient echo images massively suppressed in the spin echo versions. For example, Figure 2 shows outer volume excitations of approximately 40° for w_r_ = 0.1 that are heavily suppressed on the 2D‐FSE images (Fig. 3). This is also apparent in the images from true 3D excitation (Figs. 5 and 6). Some caution is required: unlike MLS design for gradient echo imaging (which is truly phase insensitive), the proposed relaxation for CPMG imaging provides *additional* suppression, but cannot be relied upon to fully destroy very large signals. Furthermore, this mechanism requires a long echo train to achieve good suppression; the examples given have both used trains of approximately 100 echoes with the center of k‐space encoded at the center. Use of shorter echo trains, or centric k‐space ordering would result in a lower degree of suppression, and the oscillating echo amplitudes from the non‐CPMG excitations could cause ghosting artifacts. However, the EPG simulations in Figure 1 show a factor 10 suppression in non‐CPMG echo amplitudes after only 10 echoes, suggesting shorter echo time images may also benefit from this approach. For linear order phase encoding, our results indicate that choosing a value for w_r_ ≪ 1.0 can be beneficial. The optimal choice of this parameter depends on both the design problem and the imaging sequence. Figure 2 shows that choosing w_r_ = 0 does indeed result in very low error in the CPMG component, but comes at the cost of large outer‐volume signals in the non‐CPMG part. In practice, a trade‐off is necessary, and values of approximately 0.25 were found to work well. Future work may focus on optimal choice for this parameter. An alternative to relaxing the phase as proposed would be to instead design excitation and refocusing pulses as matched pairs with fixed relative but free absolute phase 41. The rationale for using the presented method was that the refocusing pulses should remain simple and as short as possible to minimize echo spacing, with all encoding pushed to excitation, however this alternative could be an avenue for further investigation.

The small tip approximation was used for all designs. Figure 2 compares predictions of the performance of 2D spiral pulses in the linear regime and a full Bloch equation treatment. The design method achieves the desired goal of producing very low error in Im(**m**) however errors in Im(**α**) are slightly higher; this may be attributed to a failure in the STA. The discrepancy is very small; indeed, it is well known that 2D spiral trajectories are part of a “linear class” of trajectories that perform well in this regard 42. Similarly, the 3D spirals were observed to perform well empirically. For example, Figure 8 shows only a small increase in relative error in **α** compared with **m**. Linear class trajectories may be decomposed into self‐refocused components whose gradient integral is zero: this is approximately true for each turn of a 2D spiral. It is not true for each single revolution in 3D shells because these are generally offset from the origin. However it is approximately true for each individual spherical shell, and so we may conclude that 3D shells trajectories are a 3D example of a linear class trajectory, explaining the good performance achieved. Nevertheless, future work could focus on large tip angle designs to improve accuracy, potentially by means of an “additive” approach 16, 43.

Nonideal gradient performance was an issue for the high slew rate trajectories used in this work, and was overcome by the use of impulse response functions. Figure 4 shows an example of the degree of error observed. Others have proposed using measured k‐space trajectories for design 3, 44, which is convenient if using a trajectory that does not change from subject to subject 45. This work used an iterative scheme to minimize peak RF amplitudes, so in this case experimental measurement of waveforms would be cumbersome even if dedicated measurement hardware were available. Impulse response functions have been shown to perform well as a substitute 25.

3D‐FSE imaging offers a useful synergy for spatially selective excitation and nonselective refocusing pulses, and we have demonstrated effective 3D volume selection within this framework. Other work in this area has used 2D selective pulses 5, 7, 8, 9, 10. 2D selection is a natural choice for Cartesian spatial encoding, because one dimension is encoded “for free” with frequency encoding. True 3D selection does however offer one definite advantage for localization signal to a particular anatomical region: anatomy rarely conforms precisely to a 2D geometry. Even long structures (e.g., peripheral blood vessels 8 or the spine) are not rigidly one dimensional in shape, and so a curved excitation could be beneficial, particularly when signal suppression from un‐excited regions is important. The readout direction could then be chosen so as to minimize fold over of residual excited signal. Additionally, 2D excitations are often designed in a single plane using measured field properties from a single slice (or perhaps a spatial average 46) without explicit control of properties in an extended direction through plane. In reality, B_1_ and B_0_ fields are rarely invariant in this way, and so excitation quality will vary perpendicularly to the 2D excitation. A true 3D spatial design would therefore be desirable, even if a 2D trajectory is used. Finally, even in the case of a more standard 2D excitation, our results demonstrate that the proposed phase relaxation method can still be applied to improve performance.

## Supporting information


**Supporting Figure S1**. Diagram indicating the phase convention used to define the CPMG condition. a: RF pulses rotate initially z‐oriented magnetization down by flip angle |α|. b: The transverse component of the magnetization has x and y oriented components (M_x_ and M_y_). M_y_ fulfills the CPMG condition because the refocusing pulses rotate around the y‐axis (this is our convention). M_x_ violates the CPMG condition. In the complex notation used for the RF design, M_x_ corresponds to Re(α) or Re(m) while M_y_ corresponds to Im(α) or Im(m). Hence Im(α) and Im(m) are referred to as the CPMG part of the excitation, while Re(α) and Re(m) are the non‐CPMG part.
**Supporting Figure S2**. Measured impulse response function (**H**) in frequency domain for all gradient axes; the frequency resolution was 167Hz, dictated by the duration of the test waveforms (6 ms). The x and y axes are very similar, while z performance is slightly different.
**Supporting Figure S3**. The 3D shells k‐space: nominal versus predicted trajectory. Significant distortions are present.
**Supporting Figure S4**. A selection of scatter diagrams showing the relationship between two of the parameters given in Supporting Table 1, with a third represented by color coding in each case. The black triangle represents the selected trajectory. The clearest correlation is between the maximum k‐space extent (k_max_) and FWHM, which is an inverse relationship as expected. There is also a correlation between peak‐to‐sidelobe ratio (PSR) and FWHM, with small FWHM (good) tending to be associated with small PSR (bad). The selected trajectory has FWHM 21 mm and PSR 32, which part (a) suggests is a trade‐off toward the middle of both ranges. The angular offset between shells (dk_θ_) is related nonlinearly to the PSR. We see that for trajectories with small (good) FWHM, dk_θ_ = 0^°^ can yield rather small PSR. However adding some angular offset between shells allows us to increase PSR for relatively low FWHM. For this reason, dk_θ_ = 30^°^ was chosen for the selected trajectory. Overall the main impression is that while many of the parameters are interrelated, the choice of an optimum is difficult. This analysis at least provides some motivation for the selection used in the study, however it does not represent a systematic solution to the k‐space design problem.
**Supporting Figure S5**. Top: Maximum RF amplitude across all channels from the first and last iteration of the re‐VERSE design. The shaded area indicates solutions that violate the maximum amplitude constraint. Bottom: Final gradient waveforms
**Supporting Table S1**. Parameters and measured characteristics from candidate k‐space trajectories. The last column indicates the values for the selected trajectory. Under‐sample factors were computed with respect to a 220 mm cubic FOV.
