# Supplementary material for: Phase relaxed localized excitation pulses for inner volume fast spin echo imaging
Source: Magn Reson Med. 2015 Oct 9;76(3):848–61. doi: 10.1002/mrm.25996 (PMC4988531; doi:10.1002/mrm.25996)
Supplement: Supplementary file 1 — Supporting Figure S1. Diagram indicating the phase convention used to define the CPMG condition. a: RF pulses rotate initially z‐oriented magnetization down by flip angle |α|. b: The transverse component of the magnetization has x and y oriented components (Mx and My). My fulfills the CPMG condition because the refocusing pulses rotate around the y‐axis (this is our convention). Mx violates the CPMG condition. In the complex notation used for the RF design, Mx corresponds to Re(α) or Re(m) while My corresponds to Im(α) or Im(m). Hence Im(α) and Im(m) are referred to as the CPMG part of the excitation, while Re(α) and Re(m) are the non‐CPMG part. Supporting Figure S2. Measured impulse response function (H) in frequency domain for all gradient axes; the frequency resolution was 167Hz, dictated by the duration of the test waveforms (6 ms). The x and y axes are very similar, while z performance is slightly different. Supporting Figure S3. The 3D shells k‐space: nominal versus predicted trajectory. Significant distortions are present. Supporting Figure S4. A selection of scatter diagrams showing the relationship between two of the parameters given in Supporting Table 1, with a third represented by color coding in each case. The black triangle represents the selected trajectory. The clearest correlation is between the maximum k‐space extent (kmax) and FWHM, which is an inverse relationship as expected. There is also a correlation between peak‐to‐sidelobe ratio (PSR) and FWHM, with small FWHM (good) tending to be associated with small PSR (bad). The selected trajectory has FWHM 21 mm and PSR 32, which part (a) suggests is a trade‐off toward the middle of both ranges. The angular offset between shells (dkθ) is related nonlinearly to the PSR. We see that for trajectories with small (good) FWHM, dkθ = 0° can yield rather small PSR. However adding some angular offset between shells allows us to increase PSR for relatively low FWHM. For this reason, dkθ = 30° was chosen for the [file MRM-76-848-s001.docx]

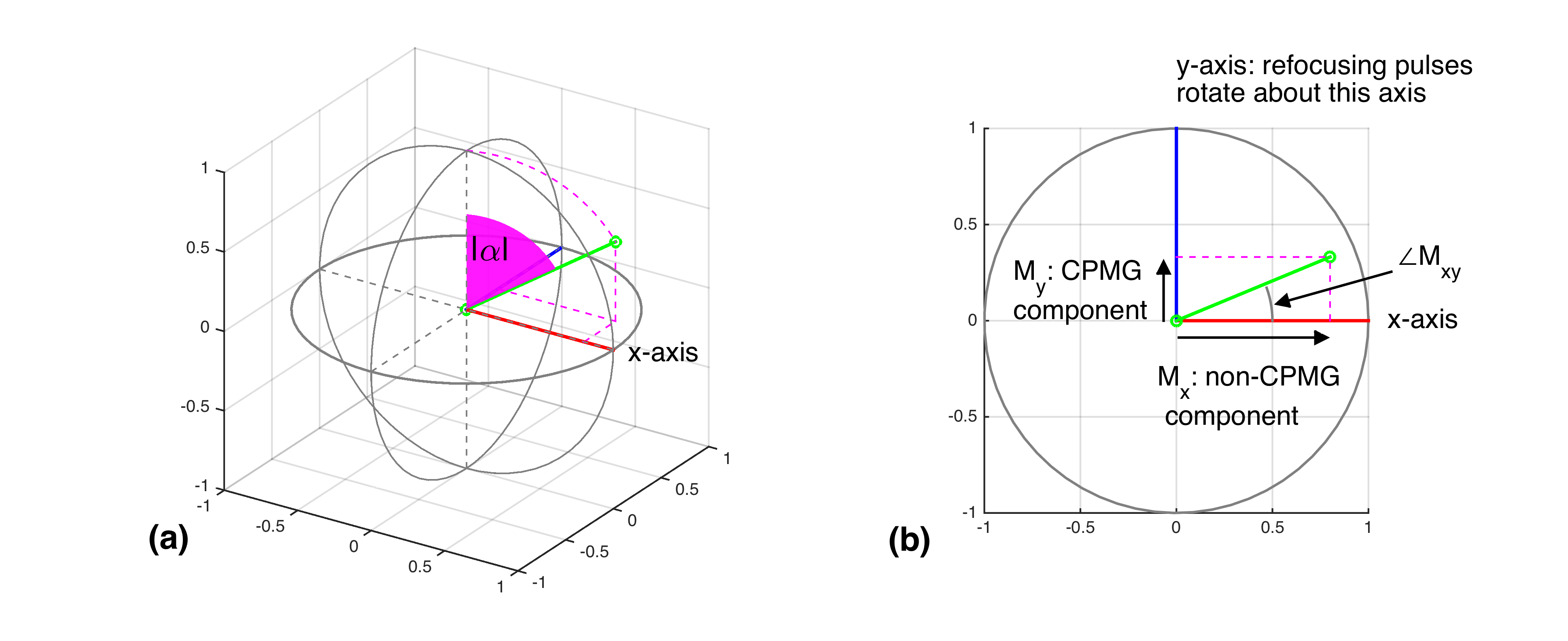


**Supporting Figure S1:** Diagram indicating the phase convention used to define the CPMG condition. (a) RF pulses rotate initially z-oriented magnetization down by flip angle |α|. (b) The transverse component of the magnetization has x and y oriented components (M_x_ and M_y_). M_y_ fulfills the CPMG condition since the refocusing pulses rotate around the y-axis (this is our convention). M_x_ violates the CPMG condition. In the complex notation used for the RF design, M_x_ corresponds to Re(α) or Re(m) while My corresponds to Im(α) or Im(m). Hence Im(α) and Im(m) are referred to as the CPMG part of the excitation, while Re(α) and Re(m) are the non-CPMG part.


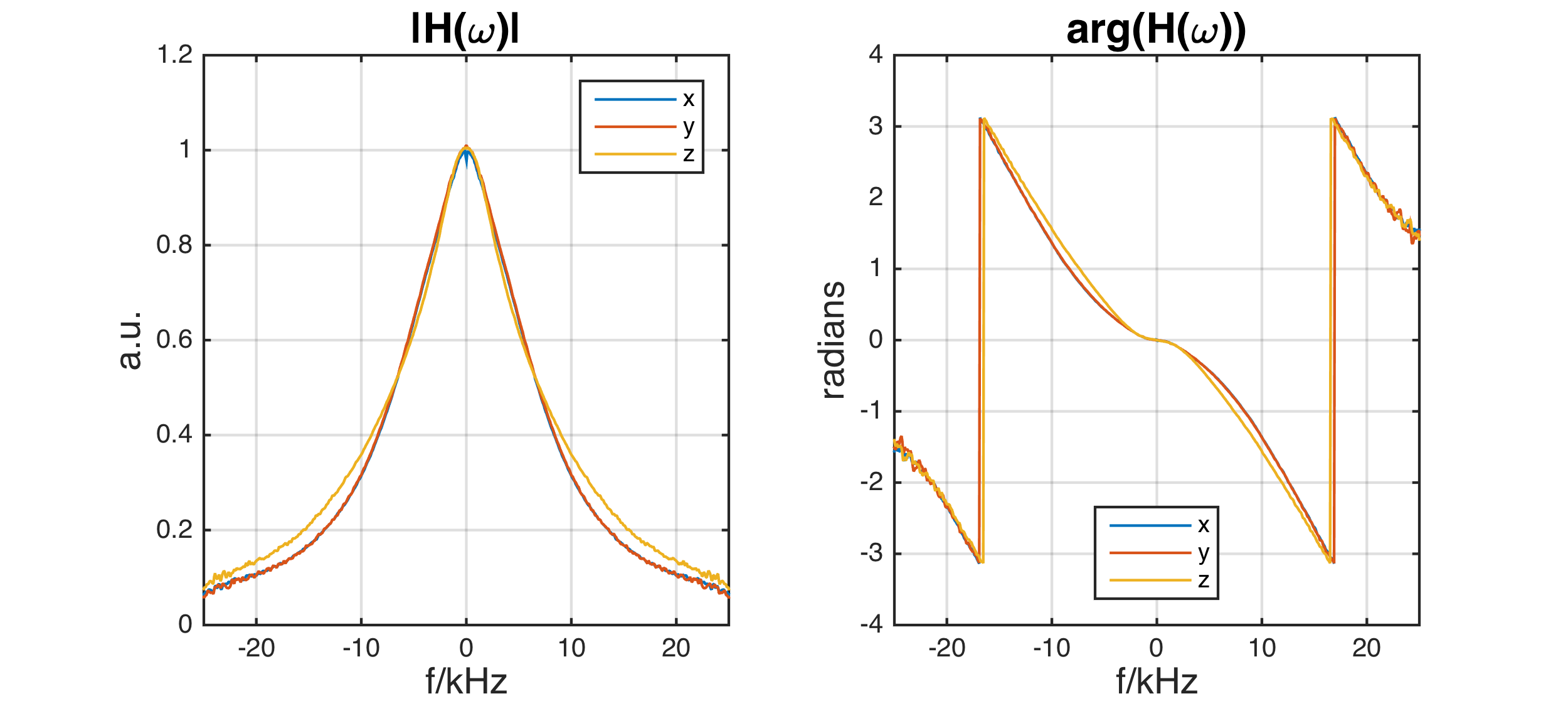


**Supporting Figure S2:** Measured impulse response function (**H**) in frequency domain for all gradient axes; the frequency resolution was 167Hz, dictated by the duration of the test waveforms (6ms). The x and y axes are very similar, while z performance is slightly different.


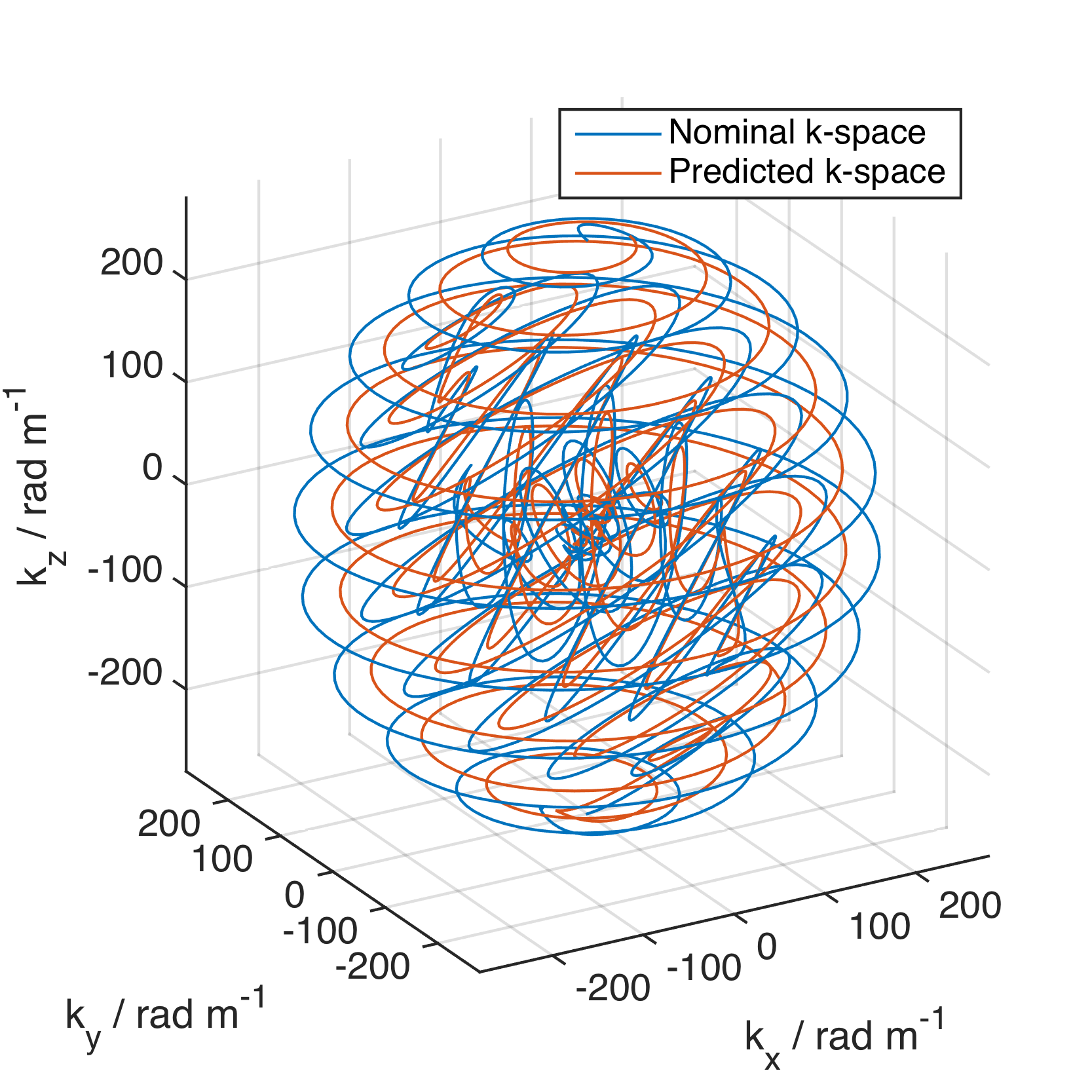


**Supporting Figure S3:** 3D shells k-space: nominal vs predicted trajectory. Significant distortions are present.


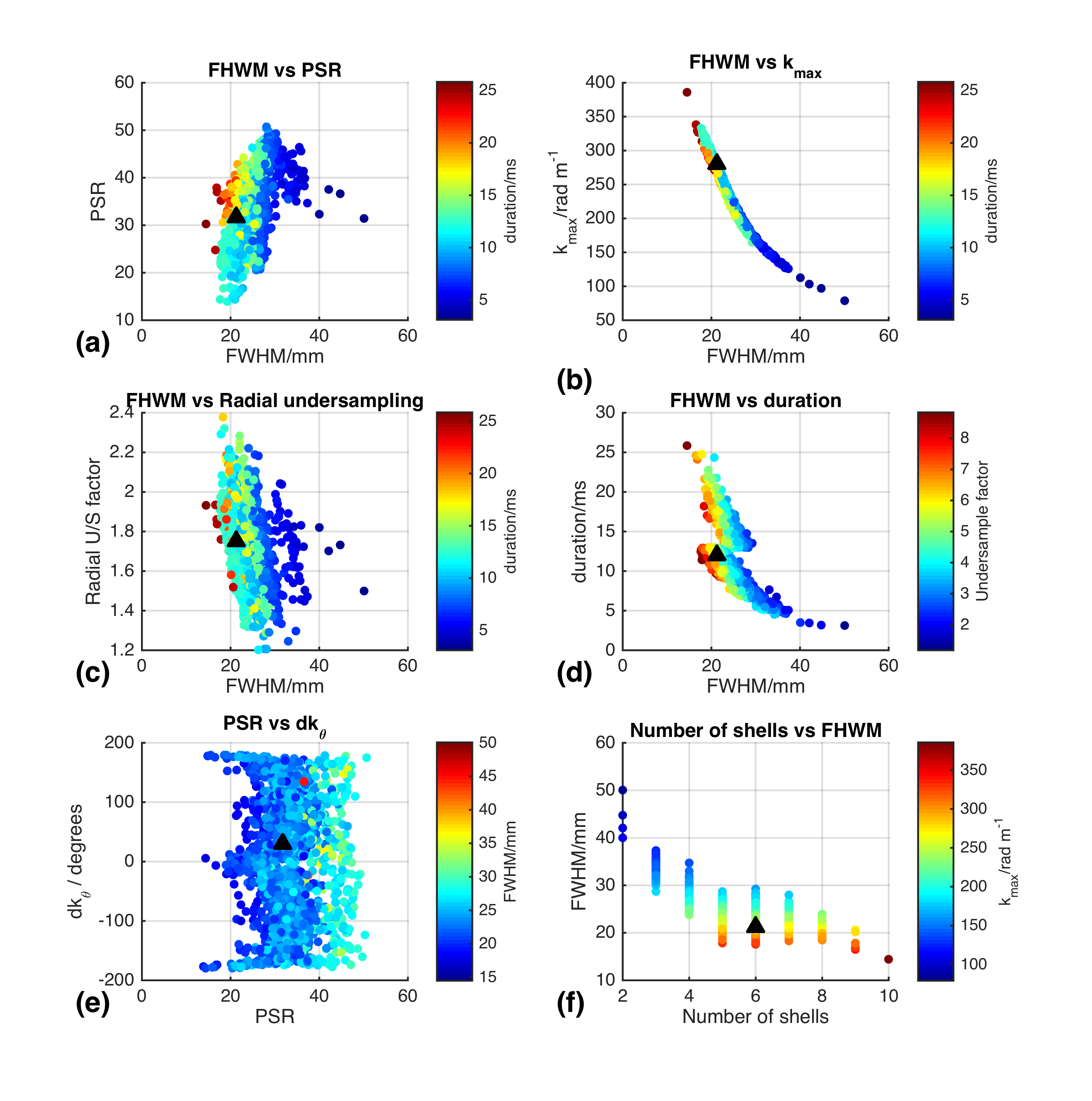


**Supporting Figure S4** A selection of scatter diagrams showing the relationship between two of the parameters given in Sup. Table 1, with a third represented by color coding in each case. The black triangle represents the selected trajectory. The clearest correlation is between the maximum k-space extent (k_max_) and FWHM, which is an inverse relationship as expected. There is also a correlation between peak to sidelobe ratio (PSR) and FWHM, with small FWHM (good) tending to be associated with small PSR (bad). The selected trajectory has FWHM 21mm and PSR 32, which part (a) suggests is a trade-off towards the middle of both ranges. The angular offset between shells (dk_θ_) is related nonlinearly to the PSR. We see that for trajectories with small (good) FWHM, dk_θ_=0° can yield rather small PSR. However adding some angular offset between shells allows us to increase PSR for relatively low FWHM. For this reason, dk_θ_=30° was chosen for the selected trajectory. Overall the main impression is that while many of the parameters are interrelated, the choice of an optimum is difficult. This analysis at least provides some motivation for the selection used in the study, however it does not represent a systematic solution to the k-space design problem.


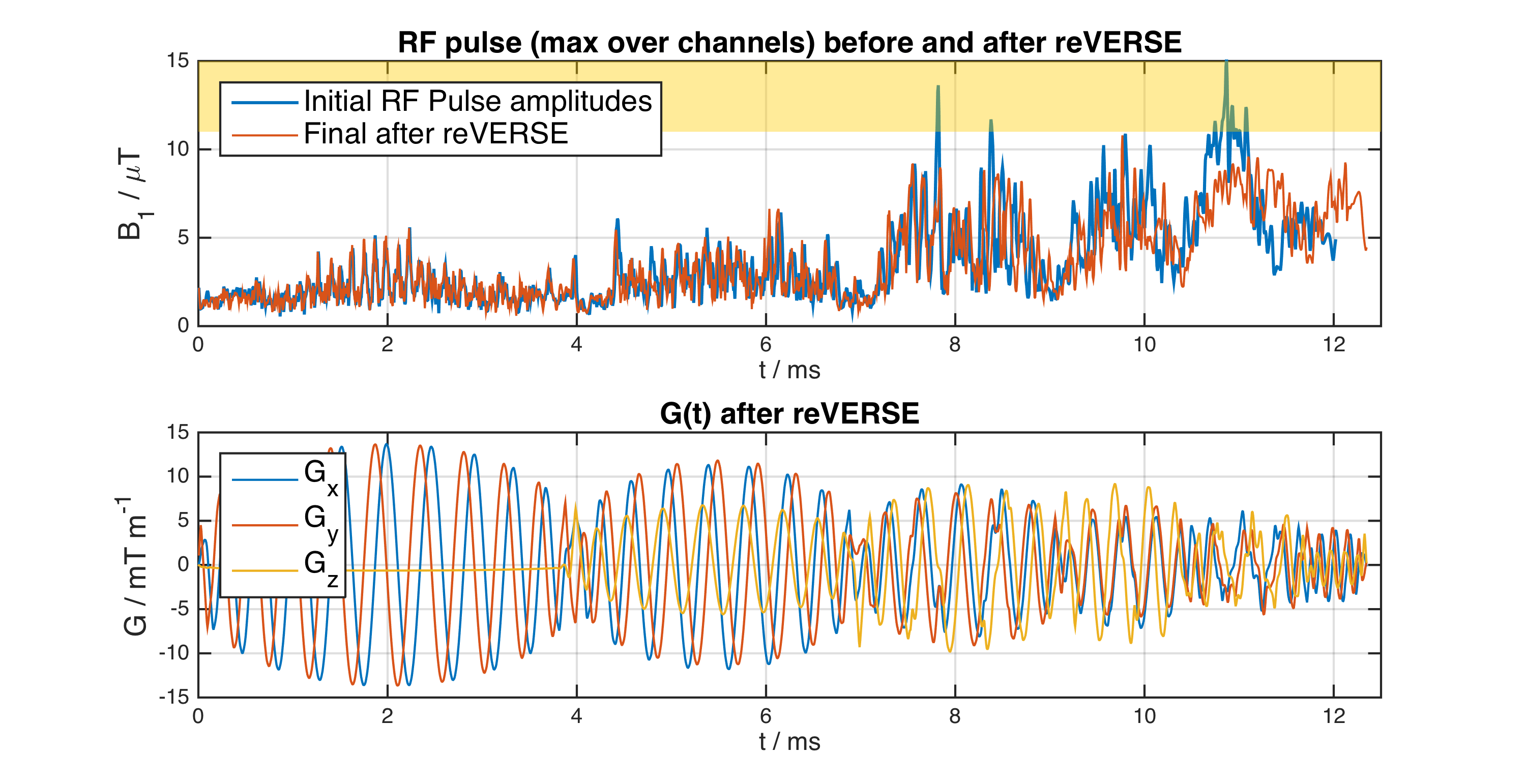


**Supporting Figure S5** Top: Maximum RF amplitude across all channels from the first and last iteration of the re-VERSE design. The shaded area indicates solutions that violate the maximum amplitude constraint. Bottom: Final gradient waveforms

| **Property** | **Mean** | **Std. Dev** | **Max** | **Min** | **Selected** |
| --- | --- | --- | --- | --- | --- |
| **Number of shells** | 5.5 | 1.1 | 10.0 | 2.0 | 6.0 |
| **K_max_ (rad m^-1^)** | 235 | 36 | 386 | 79 | 280 |
| **Radial under-sample factor** | 1.75 | 0.17 | 2.38 | 1.20 | 1.75 |
| **Angular under-sample factor** | 2.62 | 0.49 | 4.70 | 0.79 | 3.21 |
| **Overall under-sample factor** | 4.61 | 1.09 | 8.84 | 1.18 | 5.63 |
| **Duration (ms)** | 12.05 | 3.22 | 25.83 | 3.12 | 12.02 |
| **FWHM (mm)** | 24.1 | 3.0 | 50.1 | 14.5 | 21.2 |
| **PSR** | 32.9 | 5.7 | 50.7 | 13.9 | 31.7 |
| **Angular offset (degrees)** | -1.01 | 104.62 | 179.47 | -179.72 | 30.00 |

**Supporting Table S1:** Parameters and measured characteristics from candidate k-space trajectories. The last column indicates the values for the selected trajectory. Under-sample factors were computed with respect to a 220mm cubic FOV.
